# The Checking List Case Book of Dr. Maro F. Underwood

**Published:** 1896-08

**Authors:** 


### BOOK NOTICE.

THE CHECKING LIST CASE BOOK OF DR. MARO F. UNDERWOOD.

This ingenious device for studying cases was reviewed in the May number of THE HOMŒOPATHIC PHYSICIAN at page 247.

In giving the directions for using the book, it was stated at page 247, in the last paragraph, that a dash was placed opposite the name of the remedy in the letter-marked column that corresponded with the similarly lettered symptom that was being studied. This is an error. It should have said that the figure 1, 2, 3, or 4 should be placed there corresponding to the value of the remedy as given in Bœnninghausen. This, it will be seen, gives an additional advantage in getting at the remedy.

Another advantage entirely overlooked by the editor in his review is that each remedy in the book has three small figures placed over it. These figures denote, the first its antipsoric value, the second its antisycotic value, the third its antisyphilitic value.

Thus Hepar has the figures 413 over it. This signifies that as an antipsoric it has the fourth place. As an antisycotic it occupies the first place, and as an antisyphilitic it holds the third place. This also is an advantage.

